# Whole-genome sequence of an extensively drug-resistant *Acinetobacter baumannii* strain, AB/Mali-Bko/NPHI-APR.10, isolated from Bamako, Mali, in 2025

**DOI:** 10.1128/mra.01057-25

**Published:** 2026-03-13

**Authors:** Cheickna Hamallah Dicko, Ibrehima Guindo, Mohamed Ag Baraika, Ousmane Kamena, Noumou Yakhouba Keita, Demba Koita, Alhadji Dicko, Mahamadou Abdou, Klema Marcel Kone, Aissata Boubakar Cisse, Rihab Festali, Idrissa Diawara

**Affiliations:** 1 Research Laboratory of Microbiology Infectious Diseases, Allergology and Pathogen Surveillance (LARMIAS), Mohammed VI Faculty of Medicine, Mohammed VI University of Sciences and Health (UM6SS) 486625, Casablanca, Morocco; 2 Mohammed VI Higher Institute of Biosciences and Biotechnologies, Mohammed VI University of Sciences and Health (UM6SS), Casablanca, Morocco; 3 National Institute of Public Health, Bamako, Mali; 4 Faculty of Pharmacy, University of Sciences, Techniques and Technologies of Bamako (USTTB) 225803, Bamako, Mali; 5 Mohammed VI Center for Research and Innovation, Rabat, Morocco; Loyola University Chicago, Chicago, Illinois, USA

**Keywords:** Oxford Nanopore Technologies, *Acinetobacter*, genomics, Mali, WGS, bioinformatics, antibiotic resistance

## Abstract

*Acinetobacter baumannii* is a leading cause of nosocomial infections and ranks first on the World Health Organization’s list of critical-priority pathogens for antimicrobial resistance, surveillance, and new drug development. We report the whole-genome assembly of an extensively drug-resistant *Acinetobacter baumannii* from the blood of a 6-month-old child in Bamako, Mali.

## ANNOUNCEMENT

*Acinetobacter baumannii* is a major global cause of nosocomial infections, particularly carbapenem-resistant *A. baumannii* (CRAB), which are often resistant to the majority of commonly used antibiotics (1). *A. baumannii* is intrinsically resistant to many drugs and can develop resistance to nearly all antimicrobials (2, 3). Its mechanisms include β-lactamase production, efflux pumps, target-site or aminoglycoside modification, and permeability defects (3, 4). In CRAB, the predominant β-lactamases are carbapenem-hydrolyzing class D oxacillinases, which can be either acquired or intrinsic (5).

We performed whole-genome analysis of an extensively drug-resistant *A. baumannii* strain, AB/Mali-Bko/NPHI-APR.10, recovered from the blood of a 6-month-old child with suspected septicemia in Bamako, Mali. The blood culture was incubated aerobically in Brain Heart Infusion broth (Liofilchem, Italy) at 37°C for 7 days. Upon growth detection, a Gram stain was performed, followed by subculture onto Drigalski agar for 24 h at 37°C. Species identification was achieved with API 20E and confirmed using VITEK 2 Compact system, which employs advanced colorimetry for bacterial identification and integrates the Advanced Expert System for interpretive antimicrobial susceptibility testing (bioMérieux, France). The minimum inhibitory concentrations were determined using the VITEK 2 Compact AST-N240 card according to the manufacturer’s instructions. The isolate was resistant to 13 antibiotics with the MICs, as follows: ≥128 µg/mL for ticarcillin, ticarcillin-clavulanic acid, piperacillin, and piperacillin-tazobactam; ≥64 µg/mL for ceftazidime and cefepime; ≥16 µg/mL for imipenem, meropenem, gentamicin, and tobramycin; ≥4 µg/mL for ciprofloxacin; ≥8 µg/mL for levofloxacin; and ≥320 µg/mL for trimethoprim-sulfamethoxazole. The isolate was susceptible to colistin (≤0.5 µg/mL).

**Fig 1:**
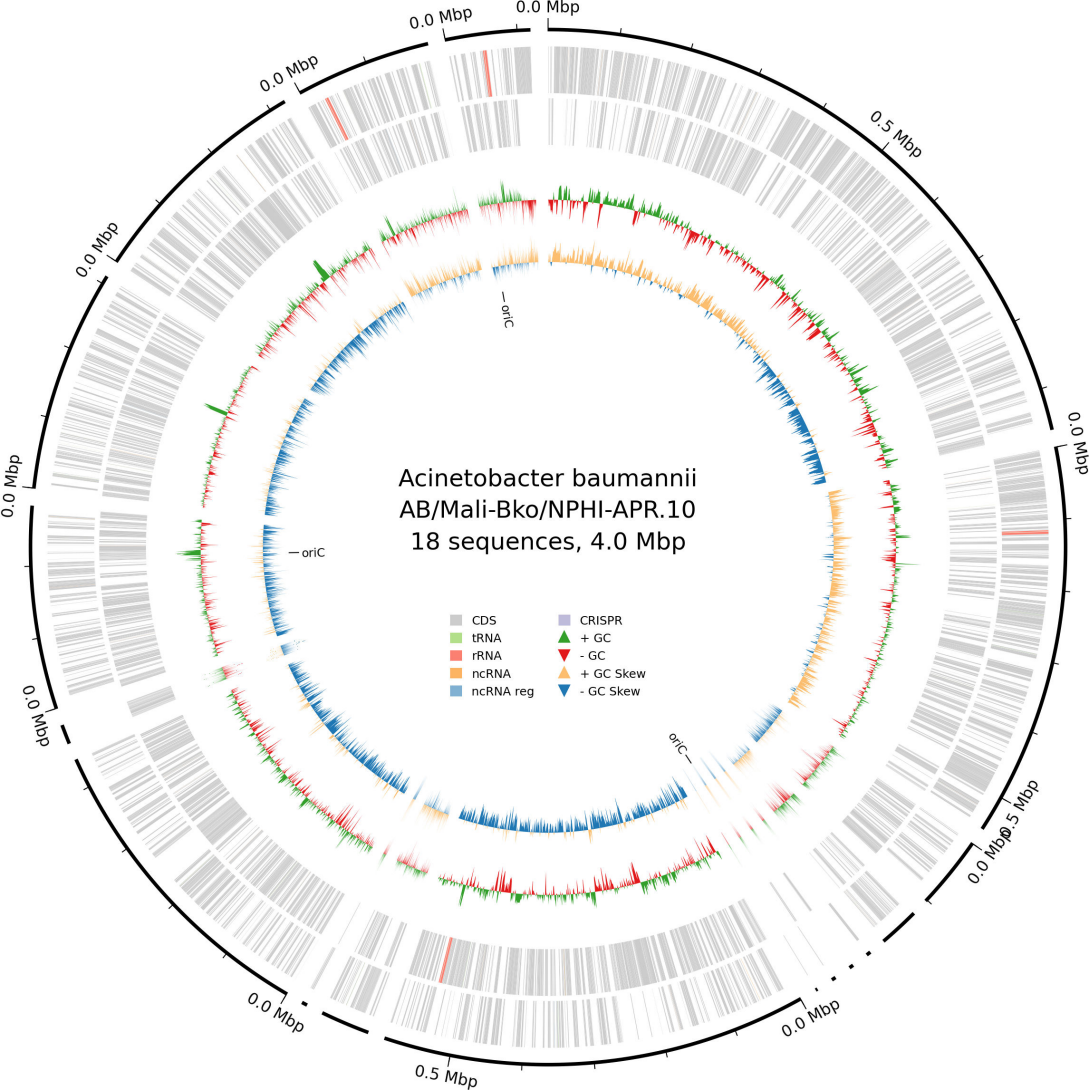
Circular genome map of the isolate. The genome comprises 18 sequences totaling 4.0 Mbp. The outer ring depicts the contigs followed by coding DNA sequences (CDS), transfer RNA (tRNA), ribosomal RNA (rRNA), non-coding RNA (ncRNA), regulatory ncRNA, and CRISPR elements on the forward and reverse strands. The middle rings show GC content (green for above and orange for below average) and GC skew (purple for positive and blue for negative), with the origin of replication (*oriC*) indicated. Genome annotation using Bakta v1.11.3 (11) reveals a compact gene organization with notable GC variation patterns typical of *A. baumannii*.

After overnight incubation on tryptic soy agar at 37°C, genomic DNA was extracted using the QIAamp Mini DNA Kit (Qiagen, USA). Genomic DNA libraries were prepared using the Rapid Barcoding Kit 96 v14 (SQK-RBK114.96; Oxford Nanopore Technologies, UK). The library was loaded on flow cell R10.4.1 (FLO-MIN114) and sequenced on minion Mk1C (Oxford Nanopore Technologies) for 72 h. High accuracy base-calling and demultiplexing were performed with Dorado v7.6.7, with a minimum read quality threshold set to 9. Read quality was assessed using FastQC v0.12.1. *De novo* assemblies were generated using Flye v2.9.5-b1801 (6) and evaluated with QUAST v5.2.0 (7). The draft genome was annotated using the Prokka pipeline v1.14.6 (8), and the publicly available genome was annotated via PGAP v6.10 (9). The isolate was assigned to ST2 via PubMLST (10). A complete integron was identified using IntegronFinder v2.0.6. Mobile genetic elements were identified using MGEfinder v1.0.3 with MGEdb v1.0.2, while plasmid detection and typing were performed using mob-suite v3.1.9. The presence of antimicrobial resistance (AMR) genes was analyzed using NCBI AMRFinderPlus v4.0.19. Table 1 presents genome data and the AMR and biocide/stress-resistance genes. A total of 43 mobile genetic elements were identified, including 33 insertion sequences, transposons, and two plasmids. Figure 1 highlights the circular genome map of the isolate. This genome provides a valuable resource for comparative genomics and phylogenetic studies of *A. baumannii*.

**TABLE 1:** Assembly metrics, NCBI accession numbers, and detected antimicrobial resistance (AMR) genes in the XDR *A. baumannii* isolate analyzed in this study

Strain (BioProject)	GenBank accession no.	Raw read data			Assembly data						No. of AMR genes^*b*^
		No. of reads	No. of bases	*N*50 (bp)	Size (bp)	Total no. of contigs	GC content (%)	*N*50 (kb)	Genome coverage	Completeness (%)^*a*^ (contamination, %)	
AB/Mali-Bko/NPHI-APR.10 (PRJNA1290668)	GCA_051414875.1	26,501	40.5 M	11,156	4,039,296	18	39	549,601	10×	85.55 (0.76)	16

^*a*^ Estimated using CheckM v1.2.4.

^*b*^ These include *bla*_*OXA-66*_, *ant(3'')-IIa*, *sul2*, *bla*_*ADC-30*_, *mph(E)*, *msr(E)*, *armA*, *sul1*, *qacEΔ1*, *aadA1*, *catB8*, *aac(6*'*)-Ib*′, *aph(3′)-Ia*, two copies of *bla*_*OXA-23*_, and *bla*_*NDM-1*_.

## Data Availability

This Whole Genome Shotgun project has been deposited at DDBJ/ENA/GenBank under accession number JBPQZJ000000000. The version described in this paper is version JBPQZJ000000000. The individual sequences are available from a hyperlink at the bottom of the WGS master record JBPQZJ000000000. The raw reads were deposited under BioProject number PRJNA1290668, Biosample number SAMN49931786, and SRA number SRR35580049.
